# Hereditary Basis of Coat Color and Excellent Feed Conversion Rate of Red Angus Cattle by Next-Generation Sequencing Data

**DOI:** 10.3390/ani12121509

**Published:** 2022-06-09

**Authors:** Yongmeng He, Yongfu Huang, Shizhi Wang, Lupei Zhang, Huijiang Gao, Yongju Zhao, Guangxin E

**Affiliations:** 1 College of Animal Science and Technology, Southwest University, Chongqing 400716, China; yongmenghe123@163.com (Y.H.); h67738337@swu.edu.cn (Y.H.); wangshizhi@swu.edu.cn (S.W.); zyongju@163.com (Y.Z.); 2 Institute of Animal Science, Chinese Academy of Agricultural Sciences (CAAS), Beijing 100193, China; zhanglupei@caas.cn (L.Z.); gaohuijiang@caas.cn (H.G.)

**Keywords:** genome-wide association study, Angus cattle, pigmentation, feed conversion rate

## Abstract

**Simple Summary:**

This study identified several variants and candidates with strong associations to coat color in Angus cattle by using genome sequencing data. In particular, the MC1R variants, which are a truncated MC1R protein obtained by altering the MCIR coding sequence region, result in lighter coat color and further enrich the genetic basis between DNA damage and melanin production caused. This study not only helped understand the hereditary basis of different coat colors, but also provided new ideas for future research on meat phenotypes. However, the putative candidate genes or markers identified in this study require further investigation to confirm their phenotypic causality and potential effective genetic relationships.

**Abstract:**

Angus cattle have made remarkable contributions to the livestock industry worldwide as a commercial meat-type breed. Some evidence supported that Angus cattle with different coat colors have different feed-to-meat ratios, and the genetic basis of their coat color is inconclusive. Here, genome-wide association study was performed to investigate the genetic divergence of black and red Angus cattle with 63 public genome sequencing data. General linear model analysis was used to identify genomic regions with potential candidate variant/genes that contribute to coat color and feed conversion rate. Results showed that six single nucleotide polymorphisms (SNPs) and two insertion–deletions, which were annotated in five genes (*ZCCHC14*, *ANKRD11*, *FANCA*, *MC1R*, and *LOC532875* [AFG3-like protein 1]), considerably diverged between black and red Angus cattle. The strongest associated loci, namely, missense mutation CHIR18_14705671 (c.296T > C) and frameshift mutation CHIR18_12999497 (c.310G>-), were located in *MC1R*. Three consecutive strongly associated SNPs were also identified and located in *FANCA*, which is widely involved in the Fanconi anemia pathway. Several SNPs of highly associated SNPs was notably enriched in *ZCCHC14* and *ANKRD11*, which are related to myofiber growth and muscle development. This study provides a basis for the use of potential genetic markers to be used in future breeding programs to improve cattle selection in terms of coat color and meat phenotype. This study is also helpful to understand the hereditary basis of different coat colors and meat phenotypes. However, the putative candidate genes or markers identified in this study require further investigation to confirm their phenotypic causality and potential effective genetic relationships.

## 1. Introduction

Angus cattle were certified in 1892 as one of the ancient British meat breeds native to Aberdeen in Northern Scotland. This breed has many excellent production performances. Its beef nutrients and meat quality occupy an irreplaceable position in the high-end beef market [1]. Many studies have been carried out on the meat quality traits [2,3], fecundity [4,5], and hybrid utilization [6] of Angus. In addition, Angus coat color (red and black) is a phenotype that can be directly observed within the breed [7]. Studies have implied that Angus cattle with red coats have better production performance (e.g., feed-to-meat and lean meat ratios) than black Angus [8]. However, the genetic basis of the coat color and economic traits of Angus remain unclear until now.

The advent of next-generation sequencing (NGS) technology has led to the confirmation of the molecular basis of a large number of phenotypes in various domestic animals [9]. Although the genetic characteristics related to the coat color and feed conversion rate of Angus cattle have not been reported, a series of genes and pathways has been widely confirmed in other cattle breeds [10,11].

In this study, the wide genomic divergence of Angus cattle with different coat colors was investigated by using a NGS dataset to explain the hereditary basis of coat color and help understand the genetic determinants that may cause differences in production performance.

## 2. Materials and Methods

The public sequencing data of 63 Angus cattle, including 21 red Angus and 42 black Angus, obtained by a previous study [12] were downloaded from the National Center for Biotechnology Information (NCBI) Sequence Read Archive Database (Table S1). High-quality reads (HQRs) were produced by filtering out reads fastp (The open-source code and corresponding instructions are available at https://github.com/OpenGene/fastp (accessed on 1 June 2021)) [13] on the basis of the https://github.com/OpenGene/fastp, custom filter parameters (cut window size 4 cut mean quality 15–5 3–3 3 length required 40). The HQRs were mapped to the cattle (*Bos taurus*) genome (ARS-UCD1.2) by using Burrows-Wheeler Aligner (version v 0.7.15) (http://bio-bwa.sourceforge.net/ (accessed on 1 June 2021)) [14], and potential duplications were removed by Picard (version v2.1.1, http://broadinstitute.github.io/picard/ (accessed on 1 June 2021)). Single nucleotide polymorphisms (SNPs) and insertion deletions (InDels) were identified and annotated by GATK Haplotype Caller [15] (version v4.1.9.0) and ANNOVAR [16]. The final VCF file was obtained using vcftools (version v0.1.16) software [17] with custom filter parameters (SNP: QD < 2.0, MQ < 40.0, FS > 60.0, SOR > 3.0, MQRankSum < −12.5, ReadPosRankSum < −8.0; Indel: QD < 2.0, FS > 200.0, SOR > 10.0, MQRankSum < −12.5, ReadPosRankSum < −8.0). Furthermore, the data quality control of SNP and InDel datasets was processed by PLINK (version v1.90b6.21, 64-bit, www.cog-genomics.org/plink/1.9/ (accessed on 1 June 2021)) and filtered by individuals with call rate >90% and minor allele frequency >0.99.

Coat color was divided into two categories. Red and black coats were as 1 and 2, respectively. For the genome-wide association study (GWAS), the SNP and InDel datasets were filtered by PLINK [18] (version v1.90b6.21, 64-bit) to exclude individuals with SNP deletion and individual deletion >0.05 and minimum allele frequency ≤0.01. The final SNP and InDel datasets for association analysis were assessed by PLINK with Chi-square test in general linear model [19]. Gene Ontology (GO) and Kyoto Encyclopedia of Genes and Genomes (KEGG) annotations were performed by KOBAS 3.0 [20]. The GO terms that were obtained were visualized using the ClueGO [21] plugin in Cytoscape (https://cytoscape.org/ (accessed on 1 June 2021)).

## 3. Results and Discussion

**Figure 1:**
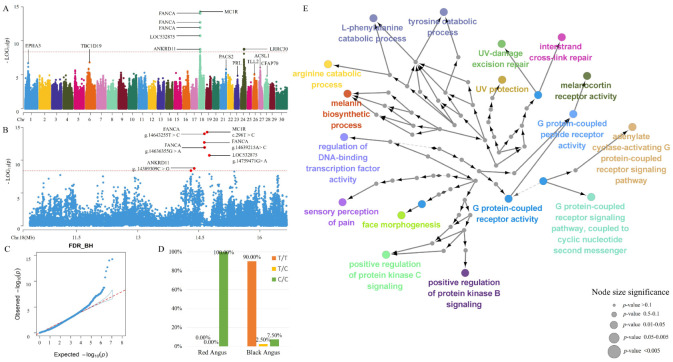
GWAS of autosomal SNPs in Angus cattle by using PLINK. (**A**) Manhattan plot of autosomal SNPs; (**B**) Manhattan plot of partial chromosome 18 SNPs (CHIR 18_10350193 to CHIR 18_16664071); (**C**) Q–Q plot of autosomal SNPs; (**D**) genotype frequencies of MC1R SNPs (c.296T> C); and (**E**) GO term enrichment of *ANKRD11*, *FANCA*, and *MC1R* as an interaction network constructed using the Cytoscape plug in ClueGO.

Before GWAS was performed, 16,797,007 SNPs and 2,563,523 InDels were obtained from the sequenced datasets of 63 Angus cattle. Among them, 11,503,619 SNPs and 1,469,221 InDels were retained after filtering using PLINK to map the locus of coat color in Angus cattle by GWAS (Figure 1). Eight SNPs exceeded the Bonferroni significant threshold (*p* < 4.3464 × 10^−9^). Among them, six SNPs were located in the exon or intron of four functional genes (*ANKRD11*, *FANCA*, *MC1R*, and *LOC532875* (AFG3-like protein 1)), and two SNPs were distributed in the intergenic sequence (Table S2). Furthermore, three of the eight SNPs were enriched in 51 GO terms, and 42 GO terms were remarkably enriched (Corrected *p* < 0.05). Seven of these GO terms were related to pigment synthesis (Table S3). The KEGG results (Table S4) revealed that two genes (*FANCA* and *MC1R*) were remarkably annotated (Corrected *p* < 0.05) in three known single pathways (Fanconi anemia (FA) pathway, melanogenesis, and neuroactive ligand–receptor interaction). The strongest association (SAM) with Corrected *p* = 5.461 × 10^−15^ was identified in CHIR18_14,705,671 (c.296T>C) in *MC1R*. In addition, two InDels (CHIR18_12999497 and CHIR18_14705684) located in *MC1R* and *ZCCHC14*, respectively, exceeded the Bonferroni significant threshold (*p* < 3.40316 × 10^−8^). These couple genes were remarkably enriched (Corrected *p* < 0.05) in 13 GO terms and two single pathways (melanogenesis and neuroactive ligand–receptor interaction Tables S5 and S6).

Previous studies verified that *MC1R* and its variant in the coding sequence region have an important and decisive influence on coat color in various mammals [22]. For example, c.844C > A (p281T> N) in cattle MC1R could cause threonine hydroxylation in melanin synthesis, resulting in the reddish coat color of cattle [23]. Both SAMs (c.296T > C and c.310G>-) from *MC1R* were already found in Asian cattle [24]. c.296T > C is a missense mutation that causes leucine (E^D^-type) to be replaced by proline (E^+^-type) at the 99th amino acid residue. Moreover, the E^D^-type *MC1R* individual remains sensitive and produces eumelanin [25]. In the present study, the results suggested that all red Angus individuals were E^+^/E^+^ homozygous, and the E^D^ gene frequency of black Angus was 0.9125 at the c.296T > C locus (Table S7).

**Figure 2:**
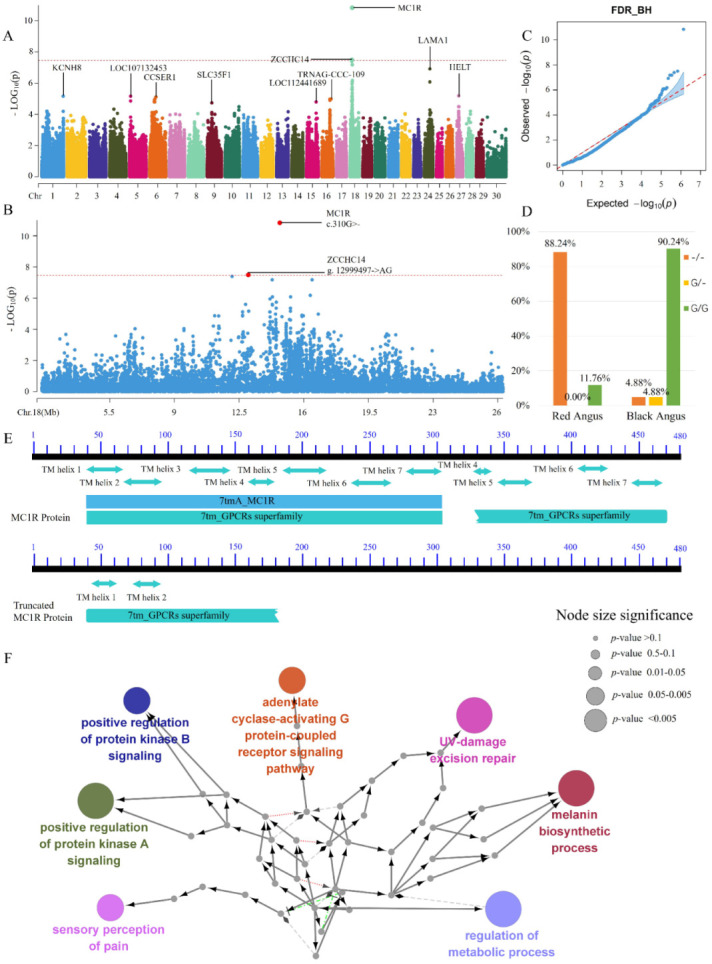
GWAS of autosomal InDels in Angus cattle by using PLINK. (**A**) Manhattan plot of autosomal InDels; (**B**) Manhattan plot of partial chromosome 18 InDels (CHIR 18_1831698 to CHIR 18_26780468); (**C**) Q–Q plot of autosomal InDels; (**D**) genotype Figure 1. *R* InDels (c.310G >–); (**E**) MC1R protein and truncated MC1R protein domains in cattle; and (**F**) GO term enrichment of *MC1R* and *ZCCHC14* as an interaction network contrasted using ClueGO.

Subsequently, a series of studies showed that the key functional structure of MC1R protein includes seven transmembrane domains (TMDs) [26]. In the present study, the gene frequency of frame-shift mutation c.310G >- had an observable difference. For example, the frequency of e-type red Angus was 0.8824, and that of E-type black Angus was 0.9268. This result is consistent with that of previous studies on the variation of MC1R in cows with different coat colors [27]. In particular, the e genotype of c.310G >- prematurely generated the stop codon because of the shift in code translation, resulting in a truncated MC1R protein with only two TMDs (Figure 2). Coincidentally, a study suggested that the truncated MC1R protein as a non-functional or weakened receptor could preferentially induce the production of phomelanin and result in red coat color [26]. Evidences supported that amino-acid variation in *MC1R* is an important genetic basis for red hair in humans [28,29].

Notably, three consecutive strongly associated SNPs (CHIR18_14636355, CHIR18_14639215, and CHIR18_14643255) were annotated to *FANCA*, which is enriched in the FA pathway. *FANCA* mutations are most commonly observed in patients with FA [30,31], and they are one of the important causes of FA. The mutant could reduce the electron transfer between respiratory complexes I–III and reactive oxygen species detoxification enzyme [32] and damage mitochondrial autophagy [33]. Its clinical features include congenital malformations and hyperpigmentation [34,35]. A previous study suggested that FANCA protein may play a role in regulating the DNA damage repair system in the FA pathway [36]. Some studies confirmed that FAP could not deal with DNA damage in the interference of DNA replication, and the α-melanocyte-stimulating hormone (α-MSH) secreted by DNA-damaged skin keratinocytes interacts with MC1R to enhance nucleotide excision repair in melanocytes [37]. Furthermore, some reports indicated a correlation between DNA damage and melanogenesis because of the function regulation of α-MSH [38].

Subsequently, a previous study stated that red Angus has a better gain-to-feed conversion rate than black Angus and that red cows are assigned to Canadian production category 1 (≥59% lean meat), whereas black cows have higher back fat thickness [8]. In the present study, a remarkable SNP (CHIR18_14759471) identified in *LOC532875* could be associated with the excellent production performance of red Angus. AFG3, as a homolog of LOC532875 and a member of the subfamily of the AAA protein family, plays an important role in mitochondrial metabolism (MM) and oxidative phosphorylation (MOP) [39,40]. Rich evidence suggested that MM and MOP have a role in muscle and fat development [41,42]. In particular, a study suggested that meat-type poultry has higher feed efficiency and faster growth than laying-type poultry because the skeletal muscle mitochondria of broilers show higher oxidative phosphorylation efficiency than those of laying chickens [43].

Moreover, two significant SNPs (CHIR18_12999497 and CHIR18_14389309) that exceeded the threshold were annotated in *ZCCHC14* and *ANKRD11*, respectively. Some studies determined that one cause of myotonic dystrophy is CCHC type zinc finger nucleic acid binding protein [44,45,46,47]. A series of widely confirmed muscle developmental genes (e.g., *FHL3*, *MYOG*, *BAG3*, *SMAD3*, and *HIF1AN*) [48], such as *MYOG*, which acts as an essential regulator of adult myofiber growth and muscle stem-cell homeostasis, interacted with *ZCCHC14* [49]. *ANKRD11* plays an important role in KBG syndrome [50]. It interacts with a series of muscle development-related genes (e.g., *HDAC3*, *HDAC4*, *SRC*, *ARRB2*, and *APEX1*) [51,52]. Which are involved in skeletal system development [53], the negative regulation of smooth muscle cell migration [54], and the positive regulation of cardiac muscle cell differentiation [55]. Coincidentally, although no direct evidence showed that *ZCCHC14*/*ANKRD11* is related to MOP, a large number of genes (e.g., *MYOD*, *SQSTM1*, and *KRAS*) that have been proven to interact with these genes display a positive regulation in oxidative phosphorylation [56,57]. Therefore, these genes are not only may be related to the development of muscle phenotype in Angus cattle and but also affects the feed conversion rate by participating in the process of oxidative phosphorylation.

Finally, another remarkably divergent SNP (CHIR24_40137470) located in the intergenic region between *LRRC30* and *LOC781276* should not be ignored. LRRC26, as a family member of LRRC, is associated with the big potassium (BK)α subunit. LRRC26 could increase the channel voltage and apparent Ca^2+^ sensitivity in arterial muscle cells to induce vasodilation and maintain the expression of functional BK channel γ subunits in muscle cells [58,59]. Furthermore, LOC781276 attracted our attention as a serine/arginine-rich splicing factor 12 (SRSF12) pseudogene. SRSF12 is involved in the regulation of mRNA selective splicing and spliceosome tri-snRNP complex assembly [60,61]. Many studies clarified that the selective RNA splicing of key gene has an irreplaceable contribution to pigmentation [62] and muscle development [63,64].

## 4. Conclusions

The coat color and feed conversion rate/lean meat ratio of domestic animals are phenotype with complex molecular basis. In this study, a series of SNPs and InDels strongly diverged between red and black Angus, as identified by GWAS method. This study could help further understand the hereditary basis of these phenotypes and support the use of available molecular markers to improve cattle breeding.

## Data Availability

The sequencing data were downloaded from the National Center for Biotechnology Information (NCBI) Sequence Read Archive Database (the accession number PRJNA176557 and PRJNA256210).

## Supplementary Materials

The following supporting information can be downloaded at: https://www.mdpi.com/article/10.3390/ani12121509/s1, Table S1. NCBI SRA information of 21 animals with red coat color and 42 animals with black coat color. Table S2. SNPs and InDels located in the exons or introns of functional genes. Table S3. Term enrichment of ANKRD11, FANCA, and MC1R. Table S4. Pathway enrichment of FANCA and MC1R. Table S5. Term enrichment of MC1R and ZCCHC14. Table S6. Pathway enrichment of MC1R. Table S7. Genotype frequencies of 10 variation sites in red and black Angus.
