# Supplementary material for: Hereditary Basis of Coat Color and Excellent Feed Conversion Rate of Red Angus Cattle by Next-Generation Sequencing Data
Source: Animals (Basel). 2022 Jun 9;12(12):1509. doi: 10.3390/ani12121509 (PMC9219544; doi:10.3390/ani12121509)
Supplement: Supplementary file 1 [file animals-12-01509-s001.zip › supplementary files/Table S1.pdf]

Table S1.NCBI SRA information of 21 animals with red coat color and 42 animals with black coat color.

Group	Code	Breed	coat color	NCBI_BioProject_ID	NCBI_BioSample_ID	NCBI_SRA_ID	Interbull_ID	Sequencing_platform	Fold_depth_after
case	1	Angus	Black	PRJNA176557	SAMN02142985	SRS428689	AANCANM000000355459	Illumina	8.28X
case	1	Angus	Black	PRJNA176557	SAMN02142982	SRS428684	AANCANM000000561205	Illumina	7.61X
case	1	Angus	Black	PRJNA176557	SAMN02142986	SRS428690	AANCANM000000564202	Illumina	8.03X
case	1	Angus	Black	PRJNA176557	SAMN02142989	SRS428688	AANCANM000001072819	Illumina	7.72X
case	1	Angus	Black	PRJNA176557	SAMN02142988	SRS428687	AANCANM000001169224	Illumina	7.89X
case	1	Angus	Black	PRJNA176557	SAMN02142990	SRS428686	AANCANM000001303204	Illumina	8.33X
case	1	Angus	Black	PRJNA176557	SAMN02142987	SRS428691	AANUSAM000002677295	Illumina	7.58X
case	1	Angus	Black	PRJNA176557	SAMN02142983	SRS428685	AANUSAM000002719761	Illumina	6.86X
case	1	Angus	Black	PRJNA176557	SAMN02143000	SRS428706	AANUSAM000008974207	Illumina	5.87X
case	1	Angus	Black	PRJNA176557	SAMN02142984	SRS428683	AANUSAM000010398616	Illumina	7.24X
case	1	Angus	Black	PRJNA176557	SAMN01915332	SRS428681	AANUSAM000012309327	Illumina	6.14X
case	1	Angus	Black	PRJNA176557	SAMN01915335	SRS428682	AANUSAM000011563543	Illumina	5.96X
case	1	Angus	Black	PRJNA176557	SAMN01915336	SRS428674	AANUSAM000011451070	Illumina	7.85X
case	1	Angus	Black	PRJNA176557	SAMN01915339	SRS428675	AANCANM000000976295	Illumina	7.59X
case	1	Angus	Black	PRJNA176557	SAMN01915337	SRS428672	AANCANM000001256842	Illumina	7.77X
case	1	Angus	Black	PRJNA176557	SAMN01915341	SRS428668	AANCANM000000912768	Illumina	7.28X
case	1	Angus	Black	PRJNA256210	SAMN02941169	SRS666774	AANUSAM000010586502	Illumina	6.37X
case	1	Angus	Black	PRJNA256210	SAMN02941170	SRS666771	AANUSAM000011261777	Illumina	6.42X
case	1	Angus	Black	PRJNA256210	SAMN02941171	SRS666769	AANUSAM000011901155	Illumina	6.83X
case	1	Angus	Black	PRJNA256210	SAMN02941172	SRS666770	AANUSAM000013207590	Illumina	6.38X
case	1	Angus	Black	PRJNA256210	SAMN02941173	SRS666692	AANUSAM000013286246	Illumina	7.14X
case	1	Angus	Black	PRJNA256210	SAMN02941174	SRS666768	AANUSAM000013936986	Illumina	6.31X
case	1	Angus	Black	PRJNA256210	SAMN02941204	SRS666767	AANUSAM000013320150	Illumina	6.26X
case	1	Angus	Black	PRJNA256210	SAMN02941205	SRS666772	AANUSAM000015148659	Illumina	6.74X
case	1	Angus	Black	PRJNA256210	SAMN02941210	SRS666773	AANCANM00ONGOLD87HC	Illumina	7.29X
case	1	Angus	Black	PRJNA176557	SAMN02843152	SRS641715	AANCANM000000631887	Illumina	17.24X
case	1	Angus	Black	PRJNA176557	SAMN02843141	SRS641713	AANCANM000001047219	Illumina	14.99X
case	1	Angus	Black	PRJNA176557	SAMN02843142	SRS641738	AANCANM000001077210	Illumina	11.36X
case	1	Angus	Black	PRJNA176557	SAMN02843143	SRS641740	AANCANM000001086586	Illumina	11.21X
case	1	Angus	Black	PRJNA176557	SAMN02843144	SRS641739	AANCANM000001504907	Illumina	11.62X
case	1	Angus	Black	PRJNA176557	SAMN02843145	SRS641732	AANCANM000001545531	Illumina	12.19X
case	1	Angus	Black	PRJNA176557	SAMN02843061	SRS629670	AANUSAM000004285114	Illumina	14.32X
case	1	Angus	Black	PRJNA176557	SAMN02142998	SRS428707	AANUSAM000006639770	Illumina	10.32X
case	1	Angus	Black	PRJNA176557	SAMN02841103	SRS629091	AANCANM000001121133	Illumina	11.57X
case	1	Angus	Black	PRJNA176557	SAMN02841106	SRS629088	AANCANM000001121114	Illumina	13.01X
case	1	Angus	Black	PRJNA176557	SAMN02841116	SRS629103	AANCANM000001413589	Illumina	11.08X
case	1	Angus	Black	PRJNA176557	SAMN02843054	SRS629688	AANCANM0000KINKA149	Illumina	11.92X
case	1	Angus	Black	PRJNA176557	SAMN02843055	SRS629690	AANCANM0000KINKA281	Illumina	11.35X
case	1	Angus	Black	PRJNA176557	SAMN02843079	SRS629677	AANCANM000001067384	Illumina	12.24X
case	1	Angus	Black	PRJNA176557	SAMN02843101	SRS631217	AANCANM000001067440	Illumina	12.36X
case	1	Angus	Black	PRJNA176557	SAMN02843131	SRS631214	AANCANM000001295406	Illumina	15.78X
case	1	Angus	Black	PRJNA176557	SAMN02839607	SRS628375	AANCANM000001267535	Illumina	11.1X
control	2	Red Angus	Red	PRJNA176557	SAMN02142999	SRS428709	RANUSAM000000013072	Illumina	5.9X
control	2	Red Angus	Red	PRJNA176557	SAMN02143001	SRS428708	RANUSAM000000832407	Illumina	5.19X
control	2	Red Angus	Red	PRJNA176557	SAMN01915333	SRS428666	RANUSAM000000372629	Illumina	7.82X
control	2	Red Angus	Red	PRJNA176557	SAMN01915334	SRS428667	RANUSAM000000574873	Illumina	7.77X
control	2	Red Angus	Red	PRJNA176557	SAMN01915350	SRS428676	RANCANM000000914772	Illumina	8.56X
control	2	Red Angus	Red	PRJNA176557	SAMN01915338	SRS428673	RANCANM000001029077	Illumina	6.93X
control	2	Red Angus	Red	PRJNA176557	SAMN01915346	SRS428664	RANCANM0000GOLD690F	Illumina	7.55X
control	2	Red Angus	Red	PRJNA176557	SAMN01915356	SRS428693	RANCANM000000967676	Illumina	7.95X
control	2	Red Angus	Red	PRJNA176557	SAMN01915359	SRS428695	RANCANM000000912788	Illumina	8.52X
control	2	Red Angus	Red	PRJNA256210	SAMN02941203	SRS666722	RANUSAM000000859497	Illumina	6.85X
control	2	Red Angus	Red	PRJNA256210	SAMN02941206	SRS666721	RANCANM000000802442	Illumina	6.32X
control	2	Red Angus	Red	PRJNA256210	SAMN02941209	SRS666708	RANCANMONGOLD7141GC	Illumina	6.61X
control	2	Red Angus	Red	PRJNA176557	SAMN02843102	SRS631196	RANCANM000000507461	Illumina	14.15X
control	2	Red Angus	Red	PRJNA176557	SAMN02843147	SRS641736	RANCANM000001016963	Illumina	13.53X
control	2	Red Angus	Red	PRJNA176557	SAMN02843122	SRS631187	RANCANM000001039064	Illumina	14.22X
control	2	Red Angus	Red	PRJNA176557	SAMN02843148	SRS641734	RANCANM000001316097	Illumina	12.72X
control	2	Red Angus	Red	PRJNA176557	SAMN02843149	SRS641737	RANCANM000001577397	Illumina	12.52X
control	2	Red Angus	Red	PRJNA176557	SAMN02843063	SRS629672	RANUSAM000000001267	Illumina	14.56X
control	2	Red Angus	Red	PRJNA176557	SAMN02842702	SRS629338	RANCANM000001379655	Illumina	11.26X
control	2	Red Angus	Red	PRJNA176557	SAMN02839613	SRS628373	RANCANM000001228327	Illumina	11.58X
control	2	Red Angus	Red	PRJNA176557	SAMN02839614	SRS628374	RANCANM000001450934	Illumina	12.48X
