# Supplementary material for: Hereditary Basis of Coat Color and Excellent Feed Conversion Rate of Red Angus Cattle by Next-Generation Sequencing Data
Source: Animals (Basel). 2022 Jun 9;12(12):1509. doi: 10.3390/ani12121509 (PMC9219544; doi:10.3390/ani12121509)
Supplement: Supplementary file 1 [file animals-12-01509-s001.zip › supplementary files/Table S2.pdf]

Table S2. SNPs and INDELs were located in exon or intron region of functional genes

Variation_Type	Chrom	Position_Info	Gene_Type	Gene_Name	*p*-values	Bonferroni corrected *p*-values
SNP	18	14389309	intronic	ANKRD11	1.096E-15	1.802E-09
SNP	18	14636355	intronic	FANCA	5.148E-20	1.974E-13
SNP	18	14639215	intronic	FANCA	1.670E-21	9.604E-15
SNP	18	14643255	intronic	FANCA	3.976E-19	1.143E-12
SNP	18	14705671	exonic	MC1R	4.747E-22	5.461E-15
SNP	18	14759471	intronic	LOC532875	8.044E-18	1.851E-11
INDEL	18	12999497	intronic	ZCCHC14	4.380E-14	3.218E-08
INDEL	18	14705684	exonic	MC1R	9.923E-18	1.458E-11

NOTE:Bonferroni significant of SNP is p<4.3464×10-9, Bonferroni significant of INDEL is p<3.40316×10-8.
