# Supplementary material for: Hereditary Basis of Coat Color and Excellent Feed Conversion Rate of Red Angus Cattle by Next-Generation Sequencing Data
Source: Animals (Basel). 2022 Jun 9;12(12):1509. doi: 10.3390/ani12121509 (PMC9219544; doi:10.3390/ani12121509)
Supplement: Supplementary file 1 [file animals-12-01509-s001.zip › supplementary files/Table S3.pdf]

Table S3. Term enrichment of ANKRD11, FANCA, and MC1R.

#Term	Ontology source	Database	ID	Input number	Background numbe	P-Value	Corrected P-Value	Input
tyrosine catabolic process	biological_process	Gene Ontology	GO:0006572	1	5	0.000458575	0.006001639	FANCA
melanocortin receptor activity	molecular_function	Gene Ontology	GO:0004977	1	5	0.000458575	0.006001639	MC1R
arginine catabolic process	biological_process	Gene Ontology	GO:0006527	1	7	0.000611403	0.006001639	FANCA
positive regulation of protein kinase (	biological_process	Gene Ontology	GO:0090037	1	10	0.000840614	0.006001639	MC1R
positive regulation of protein kinase /	biological_process	Gene Ontology	GO:0010739	1	11	0.00091701	0.006001639	MC1R
UV-damage excision repair	biological_process	Gene Ontology	GO:0070914	1	11	0.00091701	0.006001639	MC1R
L-phenylalanine catabolic process	biological_process	Gene Ontology	GO:0006559	1	11	0.00091701	0.006001639	FANCA
UV protection	biological_process	Gene Ontology	GO:0009650	1	13	0.001069791	0.006001639	MC1R
melanin biosynthetic process	biological_process	Gene Ontology	GO:0042438	1	13	0.001069791	0.006001639	MC1R
Fanconi anaemia nuclear complex	cellular_component	Gene Ontology	GO:0043240	1	14	0.001146175	0.006001639	FANCA
regulation of regulatory T cell differer	biological_process	Gene Ontology	GO:0045589	1	15	0.001222556	0.006001639	FANCA
female gonad development	biological_process	Gene Ontology	GO:0008585	1	19	0.001528039	0.006482922	FANCA
regulation of metabolic process	biological_process	Gene Ontology	GO:0019222	1	20	0.0016044	0.006482922	MC1R
tissue homeostasis	biological_process	Gene Ontology	GO:0001894	1	21	0.001680758	0.006482922	ANKRD11
male meiotic nuclear division	biological_process	Gene Ontology	GO:0007140	1	24	0.001909806	0.006551423	FANCA
regulation of DNA-binding transcript	biological_process	Gene Ontology	GO:0051090	1	26	0.002062485	0.006551423	FANCA
pigmentation	biological_process	Gene Ontology	GO:0043473	1	26	0.002062485	0.006551423	MC1R
face morphogenesis	biological_process	Gene Ontology	GO:0060325	1	31	0.002444115	0.007332346	ANKRD11
skeletal system morphogenesis	biological_process	Gene Ontology	GO:0048705	1	33	0.00259674	0.007380209	ANKRD11
G protein-coupled peptide receptor a	molecular_function	Gene Ontology	GO:0008528	1	43	0.003359631	0.00906846	MC1R
bone development	biological_process	Gene Ontology	GO:0060348	1	47	0.003664678	0.00906846	ANKRD11
negative regulation of tumor necrosis	biological_process	Gene Ontology	GO:0032720	1	49	0.003817178	0.00906846	MC1R
interstrand cross-link repair	biological_process	Gene Ontology	GO:0036297	1	50	0.003893423	0.00906846	FANCA
sensory perception of pain	biological_process	Gene Ontology	GO:0019233	1	53	0.004122132	0.00906846	MC1R
odontogenesis of dentin-containing t	biological_process	Gene Ontology	GO:0042475	1	57	0.004427024	0.009194588	ANKRD11
G protein-coupled receptor signaling	biological_process	Gene Ontology	GO:0007187	1	65	0.005036621	0.010073241	MC1R
regulation of inflammatory response	biological_process	Gene Ontology	GO:0050727	1	82	0.006331187	0.012072566	FANCA
multicellular organism growth	biological_process	Gene Ontology	GO:0035264	1	84	0.006483415	0.012072566	ANKRD11
male gonad development	biological_process	Gene Ontology	GO:0008584	1	93	0.007168249	0.012902849	FANCA
adenylate cyclase-activating G protei	biological_process	Gene Ontology	GO:0007189	1	113	0.008688976	0.014662646	MC1R
protein-containing complex assembly	biological_process	Gene Ontology	GO:0065003	1	129	0.009904438	0.015971772	FANCA
anatomical structure morphogenesis	biological_process	Gene Ontology	GO:0009653	1	131	0.010056301	0.015971772	ANKRD11
positive regulation of protein kinase E	biological_process	Gene Ontology	GO:0051897	1	166	0.012711389	0.01940812	MC1R
regulation of cell population prolifera	biological_process	Gene Ontology	GO:0042127	1	169	0.012938747	0.01940812	FANCA
in utero embryonic development	biological_process	Gene Ontology	GO:0001701	1	202	0.015437379	0.022530229	ANKRD11
DNA repair	biological_process	Gene Ontology	GO:0006281	1	224	0.017100789	0.024301122	FANCA
ubiquitin protein ligase binding	molecular_function	Gene Ontology	GO:0031625	1	294	0.022380992	0.030989065	MC1R
nucleoplasm	cellular_component	Gene Ontology	GO:0005654	2	3630	0.024101823	0.032537462	FANCA|ANKRD11
intracellular signal transduction	biological_process	Gene Ontology	GO:0035556	1	369	0.02801733	0.036022281	MC1R
multicellular organism development	biological_process	Gene Ontology	GO:0007275	1	462	0.034976249	0.043923662	MC1R
plasma membrane	cellular_component	Gene Ontology	GO:0005886	2	4619	0.038318586	0.046077628	MC1R|ANKRD11
cytoplasm	cellular_component	Gene Ontology	GO:0005737	2	4624	0.038398023	0.046077628	MC1R|FANCA
cytosol	cellular_component	Gene Ontology	GO:0005829	2	5095	0.046211289	0.054248034	FANCA|ANKRD11
nucleus	cellular_component	Gene Ontology	GO:0005634	2	5208	0.048181742	0.055357746	FANCA|ANKRD11
G protein-coupled receptor activity	molecular_function	Gene Ontology	GO:0004930	1	739	0.055506397	0.062444696	MC1R
G protein-coupled receptor signaling	biological_process	Gene Ontology	GO:0007186	1	1132	0.084131323	0.092716152	MC1R
positive regulation of transcription by	biological_process	Gene Ontology	GO:0045944	1	1159	0.086076403	0.092962515	MC1R
integral component of plasma memb	cellular_component	Gene Ontology	GO:0005887	1	1380	0.10189382	0.107887574	MC1R
extracellular exosome	cellular_component	Gene Ontology	GO:0070062	1	2085	0.151130524	0.156943236	FANCA
metal ion binding	molecular_function	Gene Ontology	GO:0046872	1	2298	0.165644096	0.168769457	FANCA
protein binding	molecular_function	Gene Ontology	GO:0005515	2	11779	0.216215139	0.216215139	MC1R|FANCA
