# Supplementary material for: Hereditary Basis of Coat Color and Excellent Feed Conversion Rate of Red Angus Cattle by Next-Generation Sequencing Data
Source: Animals (Basel). 2022 Jun 9;12(12):1509. doi: 10.3390/ani12121509 (PMC9219544; doi:10.3390/ani12121509)
Supplement: Supplementary file 1 [file animals-12-01509-s001.zip › supplementary files/Table S4.pdf]

Table S4.Pathway enrichment of FANCA and MC1R.

KEGG I	KEGG II	KEGG III	Database	ID	Input number	Background number	P-Value	Corrected P-Valu	Gene-name
Genetic Information Processing	Replication and repair	Fanconi anemia pathway	KEGG PATHWAY	hsa03460	1	54	0.004198361	0.00906846	FANCA
Organismal Systems	Endocrine system	Melanogenesis	KEGG PATHWAY	hsa04916	1	101	0.007776726	0.013546555	MC1R
Environmental Information Processing	Signaling molecules and interaction	Neuroactive ligand-receptor interaction	KEGG PATHWAY	hsa04080	1	338	0.025690277	0.033835975	MC1R
