# Supplementary material for: Hereditary Basis of Coat Color and Excellent Feed Conversion Rate of Red Angus Cattle by Next-Generation Sequencing Data
Source: Animals (Basel). 2022 Jun 9;12(12):1509. doi: 10.3390/ani12121509 (PMC9219544; doi:10.3390/ani12121509)
Supplement: Supplementary file 1 [file animals-12-01509-s001.zip › supplementary files/Table S5.pdf]

Table S5.Term enrichment of MC1R and ZCCHC14.

Ontology source	#Term	Database	ID	Input number	Background	P-Value	Corrected P-Value	gene_name
biological_process	positive regulation of protein kinase A signaling	Gene Ontology	GO:0010739	1	6	0.000483793	0.003991016	MC1R
biological_process	positive regulation of protein kinase C signaling	Gene Ontology	GO:0090037	1	6	0.000483793	0.003991016	MC1R
biological_process	UV-damage excision repair	Gene Ontology	GO:0070914	1	10	0.000760193	0.003991016	MC1R
biological_process	melanin biosynthetic process	Gene Ontology	GO:0042438	1	10	0.000760193	0.003991016	MC1R
biological_process	regulation of metabolic process	Gene Ontology	GO:0019222	1	18	0.00131288	0.004836842	MC1R
biological_process	pigmentation	Gene Ontology	GO:0043473	1	19	0.001381955	0.004836842	MC1R
biological_process	sensory perception of pain	Gene Ontology	GO:0019233	1	33	0.002348755	0.006165482	MC1R
biological_process	negative regulation of tumor necrosis factor production	Gene Ontology	GO:0032720	1	33	0.002348755	0.006165482	MC1R
molecular_function	phosphatidylinositol binding	Gene Ontology	GO:0035091	1	68	0.004763706	0.010278967	ZCCHC14
biological_process	positive regulation of protein kinase B signaling	Gene Ontology	GO:0051897	1	73	0.00510846	0.010278967	MC1R
biological_process	adenylate cyclase-activating G protein-coupled receptor signali	Gene Ontology	GO:0007189	1	77	0.005384221	0.010278967	MC1R
molecular_function	ubiquitin protein ligase binding	Gene Ontology	GO:0031625	1	213	0.014737329	0.023806455	MC1R
molecular_function	nucleic acid binding	Gene Ontology	GO:0003676	1	235	0.01624618	0.02436927	ZCCHC14
molecular_function	zinc ion binding	Gene Ontology	GO:0008270	1	664	0.045437673	0.05612889	ZCCHC14
molecular_function	G protein-coupled receptor activity	Gene Ontology	GO:0004930	1	664	0.045437673	0.05612889	MC1R
biological_process	positive regulation of transcription by RNA polymerase II	Gene Ontology	GO:0045944	1	872	0.059432864	0.069338341	MC1R
cellular_component	plasma membrane	Gene Ontology	GO:0005886	1	2339	0.155204299	0.171541593	MC1R
cellular_component	cytoplasm	Gene Ontology	GO:0005737	1	2867	0.188415995	0.197836795	MC1R
cellular_component	integral component of membrane	Gene Ontology	GO:0016021	1	3697	0.239277463	0.239277463	MC1R
