# Supplementary material for: Hereditary Basis of Coat Color and Excellent Feed Conversion Rate of Red Angus Cattle by Next-Generation Sequencing Data
Source: Animals (Basel). 2022 Jun 9;12(12):1509. doi: 10.3390/ani12121509 (PMC9219544; doi:10.3390/ani12121509)
Supplement: Supplementary file 1 [file animals-12-01509-s001.zip › supplementary files/Table S6.pdf]

Table S6.Pathway enrichment of MC1R.

KEGG I	KEGG II	KEGG III	Database	ID	Input numbe	Background nu	P-Value	Corrected P-Value	gene_name
Organismal Systems	Endocrine system	Melanogenesis	KEGG PATHWAY	bta04916	1	102	0.007106857	0.012436999	MC1R
Environmental Information Processing	Signaling molecules and interaction	Neuroactive ligand-receptor interaction	KEGG PATHWAY	bta04080	1	363	0.025002016	0.035002822	MC1R
