# Supplementary material for: Hereditary Basis of Coat Color and Excellent Feed Conversion Rate of Red Angus Cattle by Next-Generation Sequencing Data
Source: Animals (Basel). 2022 Jun 9;12(12):1509. doi: 10.3390/ani12121509 (PMC9219544; doi:10.3390/ani12121509)
Supplement: Supplementary file 1 [file animals-12-01509-s001.zip › supplementary files/Table S7.pdf]

Table S7. Genotype frequencies of 10 variation sites in red and black Angus.

varint type	chr	site	gene	location type	RED_ANGUS						BLACK_ANGUS					
					AA	%	AB	%	BB	%	AA	%	AB	%	BB	%
snp	18	14306763	SLC22A31(dist=24574),gene-ANKRD11(dist=20083)	intergenic	9	47.37%	7	36.84%	3	15.79%	0	0.00%	1	2.44%	40	97.56%
snp	18	14389309	ANKRD11	intronic	11	64.71%	4	23.53%	2	11.76%	2	4.76%	1	2.38%	39	92.86%
snp	18	14636355	FANCA	intronic	16	84.21%	2	10.53%	1	5.26%	2	4.76%	1	2.38%	39	92.86%
snp	18	14639215	FANCA	intronic	17	89.47%	2	10.53%	0	0.00%	2	4.76%	1	2.38%	39	92.86%
snp	18	14643255	FANCA	intronic	16	88.89%	2	11.11%	0	0.00%	3	7.14%	2	4.76%	37	88.10%
snp	18	14705671	MC1R	exonic	0	0.00%	0	0.00%	20	100.00%	36	90.00%	1	2.50%	3	7.50%
snp	18	14759471	LOC532875	intronic	13	68.42%	5	26.32%	1	5.26%	1	2.38%	2	4.76%	39	92.86%
snp	24	40137470	LRRC30(dist=8121),gene-LOC781276(dist=72289)	intergenic	10	50.00%	5	25.00%	5	25.00%	0	0.00%	0	0.00%	41	100.00%
indel	18	12999497	ZCCHC14	intronic	12	57.14%	7	33.33%	2	9.52%	2	4.76%	3	7.14%	37	88.10%
indel	18	14705685	MC1R	exonic	15	88.24%	0	0.00%	2	11.76%	2	4.88%	2	4.88%	37	90.24%
